# Hospital value-based payment programs and disparity in the United States: A review of current evidence and future perspectives

**DOI:** 10.3389/fpubh.2022.882715

**Published:** 2022-10-10

**Authors:** Hyunmin Kim, Asos Mahmood, Noah E. Hammarlund, Cyril F. Chang

**Affiliations:** ^1^School of Health Professions, The University of Southern Mississippi, Hattiesburg, MS, United States; ^2^Division of Health Systems Management and Policy, School of Public Health, The University of Memphis, Memphis, TN, United States; ^3^Center for Health System Improvement, College of Medicine, University of Tennessee Health Science Center, Memphis, TN, United States; ^4^Department of Medicine-General Internal Medicine, College of Medicine, University of Tennessee Health Science Center, Memphis, TN, United States; ^5^Department of Health Services Research, Management and Policy, University of Florida, Gainesville, FL, United States; ^6^Department of Economics, Fogelman College of Business and Economics, The University of Memphis, Memphis, TN, United States

**Keywords:** Value-Based Purchasing, Medicare, health equity, payment reform, upstream approach, hospital VBP, health care disparity, social determinant of health

## Abstract

Beginning in the early 2010s, an array of Value-Based Purchasing (VBP) programs has been developed in the United States (U.S.) to contain costs and improve health care quality. Despite documented successes in these efforts in some instances, there have been growing concerns about the programs' unintended consequences for health care disparities due to their built-in biases against health care organizations that serve a disproportionate share of disadvantaged patient populations. We explore the effects of three Medicare hospital VBP programs on health and health care disparities in the U.S. by reviewing their designs, implementation history, and evidence on health care disparities. The available empirical evidence thus far suggests varied impacts of hospital VBP programs on health care disparities. Most of the reviewed studies in this paper demonstrate that hospital VBP programs have the tendency to exacerbate health care disparities, while a few others found evidence of little or no worsening impacts on disparities. We discuss several policy options and recommendations which include various reform approaches and specific programs ranging from those addressing upstream structural barriers to health care access, to health care delivery strategies that target service utilization and health outcomes of vulnerable populations under the VBP programs. Future studies are needed to produce more explicit, conclusive, and consistent evidence on the impacts of hospital VBP programs on disparities.

## Background and introduction

There have been numerous recent efforts in the United States (U.S.) to improve the quality of hospital care and contain costs. For years, American hospitals were reimbursed under a cost-based fee-for-service (FFS) system (1, 2). Though not the sole cause of all the financial and quality of care issues troubling the U.S. hospital system (3), the volume-driven and cost-based FFS payment system emerged as both a symbol of root cause and a target of reform. In the 1980s, for example, Medicare led the way in hospital payment reform by paying hospitals a fixed and predetermined amount based on a patient's admitting diagnosis and how it was classified in a new patient classification system called the diagnosis-related group (DRG). Today, the DRG system is still how Medicare reimburses hospitals, and private payers and health plans have all adopted some variant of this hospital payment model.

The DRG system is not without its own limitations and drawbacks. On the one hand, for example, hospital costs continued to grow rapidly, albeit at a slightly slower pace, after the adoption of the DRG system. On the other hand, the twin issues of quality of care and the growing inequality of care became the focus of reform beginning in early 2000 as DRG's lack of attention to quality and inequality became a pressing issue. Most recently in the first decade of the twenty-first century, a new class of payment and delivery models was developed and promoted to use patient and provider incentives to improve quality and to slow down the growth of health care expenditures (4). These included such new payment models as bundled payments that involved fixed and predetermined payments for delivering an entire episode of hospital care, Accountable Care Organizations (ACOs) that comprised providers and hospitals working together to deliver coordinated care, and patient-centered medical homes (PCMH) that provided coordinated care in the primary care settings to avoid expensive hospital care. In addition, and most importantly, a new class of pay-for-performance (P4P) programs that rewarded hospitals for adhering to accepted and evidence-based quality standards and processes were adopted under the general heading of the Value-Based Purchasing (VBP) Programs.

The purpose of this paper is to conduct a review of literature published between 2012 and 2022 to examine three original Centers for Medicare and Medicaid Service's (CMS) VBP programs that are hospital based: Hospital Value-Based Purchasing Program (HVBP), Hospital Readmission Reduction Program (HRRP), and Hospital-Acquired Condition Reduction Program (HACRP). Given that hospital care accounts for the largest portion of the U.S. total health expenditure (about 33%), and Medicare being the second largest source of it (5), it is urgent that we assess these programs' successes and pitfalls. Our specific aim is to evaluate the strength of evidence, both theoretical and empirical, that links the observed disparities in access and outcomes to the value-based method of reimbursing hospital care initiated by the CMS. We defined disparities as differences in quality and outcomes between groups of people that are closely linked to their social, economic, and environmental disadvantages over and beyond those that are attributable to variations in health care needs, patient preferences, and evidence-based professional treatment recommendations (6–8). The study contributes to the existing literature by filling a gap in our understanding of the implications of hospital VBP as a payment model on health and health care disparities and in opening future dialogues for improving health equity.

### CMS's hospital VBP programs

The CMS has a long history of using financial incentives to improve quality and save costs. As early as the 1980s, for example, the CMS, or the federal Health Care Financing Administration (HCFA) as it was then called, began in earnest to explore the principles and mechanisms such as capitation and prospective payments to slow the growth of total as well as per capita Medicare and Medicaid costs. Specifically, in 1983, the DRG method of payment began, shifting from an FFS-based reimbursement to a lump sum payment to hospitals based on the patient's hospital admission and diagnoses. Although this payment contributed to lowering the growth rate of Medicare hospital costs, it was not sufficient to control rising costs associated with Medicare outpatient services and those brought by private purchasers (9).

In the early years of the Patient Protection and Affordable Care Act (ACA), the CMS, under the authorization of Congress and the urging of the Obama Administration, initiated a wide array of hospital VBP programs (i.e., HVBP, HRRP, and HACRP) to promote reimbursements that reward quality rather than volume. Both HVBP and HRRP were first enforced in 2012, and the HACRP was implemented in 2014. The Hospital-based VBP programs were built on the infrastructure of the Premier Hospital Quality Incentive Demonstration (HQID), a hospital P4P demonstration model, that was first implemented by the CMS in 2003 (10–12). The HQID used quality of inpatient care as the bases of providing hospitals with incentives (12). After the ACA, hospital-based VBP model expanded greatly upon the HQID and even employed several of the same performance measures in its design (12). As a matter of policy and program evaluation, the U.S. Department of Health and Human Services (DHHS) declared in 2015 that these programs would tie 85% of all Medicare FFS payments to value or quality by 2016 and 90% by 2018 (13).

In the HVBP Program, payment adjustment is made by a total performance score (TPS) based on quality compared to all hospitals, or on how much improvement hospitals have made on their own (14). Notably, quality is evaluated based on quality domains such as the process of care, clinical outcomes, safety, and patient experience (15). Further, the HVBP program offers favorable chances for extra payments to hospitals which reach performance thresholds or those which accomplish significant improvement in domains compared to the basic standard. In 2013 and 2014, it was shown that the largest amounts of extra, and penalty, payments were 1.25% of Medicare inpatient payments (14). Meanwhile, in the HACRP Program, quality assessment is carried out by calculating a score of total hospital-acquired conditions of which a value of 75% and above on the score scale will have reduced Medicare payments while payment reduction is reassessed by reflecting on the overall Medicare payments subject to other value-based payments (16, 17).

The HRRP, starting in 2012, assesses the quality of outcomes using the excessive readmission ratio (ERR) (i.e., predicted-to-expected readmissions) for the following conditions/procedures: heart failure (HF), pneumonia (with its additional types), acute myocardial infarction (AMI), chronic obstructive pulmonary disease, elective primary total hip arthroplasty and/or total knee arthroplasty, and coronary artery bypass graft surgery (18). A penalty is imposed on hospitals with ERRs larger than one, whereas those with an ERR of one or less are not subject to penalization. The penalty was initially limited to 1% of DRG payments. Then the payment limit increased to 2% in 2014 and 3% in 2015, in which CMS caps penalties at 3%. With the enactment of the twenty-first Century Cures Act in 2016, a change was to be made to HRRP in 2019 by adjusting for social risk factors through peer grouping to address a concern about its penalization of hospitals serving patients with socioeconomic disadvantages (19, 20). That is, based on the percentage of a hospital's dual Medicare-Medicaid inpatients, hospitals are categorized into one of five peer groups (20), of which those that exceed the median readmission rates (after controlling for risk factors) are penalized (21). Previously, the HRRP's formula for social risk adjustment had a drawback that it did not adequately control for social risk factors among patients resulting in higher readmissions (22) against which the peer grouping approach could address the concern. An overview of the three hospital VBP programs is provided in Table 1.

**Table 1:** Medicare hospital-based VBP programs in the United States.

**Medicare hospital VBP programs**	**Brief overview**	**First year of implementation**	Included measures*
Hospital Value-Based Purchasing Program	Improve quality outcomes, such as mortality, healthcare-related infections, efficiency, etc. in acute care hospitals by adjusting Medicare payments	FY 2013	Patient safety measures: • CLABSI • CAUTI • SSI for colon surgeries and abdominal hysterectomies • MRSA bacteremia • CDI Clinical outcomes: • 30-day mortality rates for: AMI, COPD, HF, and pneumonia • THA/TKA complication rate • Efficiency and cost reduction: • MSPB Person and community engagement (HCAHPS survey): • Communication with doctors • Communication with nurses • Responsiveness of hospital staff • Communication about medicines • Hospital cleanliness and quietness • Discharge information • Care transition • Overall rating of hospital
Hospital readmission reduction program	Reduce hospital readmission rates by enhancing care coordination and communication, assessed by the excessive readmission ratio	FY 2013	•AMI •HF •COPD •Pneumonia •CABG surgery •THA/TKA
Hospital acquired conditions reduction program	Engage acute care hospitals to adopt best practices to reduce hospital-acquired infections to improve patient safety and outcomes	FY 2015	Patient safety and adverse events composite (CMS PSI 90): • PSI 03: Pressure ulcer rate • PSI 06: Iatrogenic pneumothorax Rate • PSI 08: In hospital fall with hip fracture rate • PSI 09: Perioperative hemorrhage or hematoma rate • PSI 10: Post-operative acute kidney injury requiring dialysis rate • PSI 11: Post-operative respiratory failure rate • PSI 12: Perioperative pulmonary embolism or deep vein thrombosis rate • PSI 13: Post-operative sepsis rate • PSI 14: Post-operative wound dehiscence rate • PSI 15: Abdominopelvic accidental puncture or laceration rate CDC NHSN HAI measures: • CLABSI • CAUTI • SSI for colon surgeries and abdominal hysterectomies • MRSA bacteremia • CDI

AMI, Acute myocardial infarction; CABG, Coronary artery bypass graft; CAUTI, Catheter-Associated urinary tract infection; CDC, Centers for disease control and prevention; CDI, Clostridium difficile infection; CLABSI, Central line-associated bloodstream infection; CMS, Centers for medicare & medicaid services; COPD, Chronic obstructive pulmonary disease; FY, Federal fiscal year; HAI, Hospital-Acquired infection; HCAHPS, Hospital consumer assessment of healthcare providers and systems; HF, Heart failure; MRSA, Methicillin-Resistant Staphylococcus aureus; MSPB, Medicare spending per beneficiary; NHSN, National healthcare safety network; PSI, Patient safety indicator; SSI, Surgical site infection; THA/TKA, Elective primary total hip arthroplasty and/or total knee arthroplasty; VBP, Value-Based payment.

^*^As of federal fiscal year 2022.

Sources: Centers for Medicare & Medicaid Services and The U.S. Department of Health & Human Services (14–18).

### Hospital VBP effectiveness and implications for equity and health care disparities

To date, available evidence on whether hospital-based VBP programs achieved their primary quality and cost reduction goals is mixed (12, 23). Many studies have found improvements in health care quality in certain cases even though the effects on cost containment appear to have been modest (4, 24–26). Other studies found no improvement in quality measures and claimed increased costs for certain programs and conditions (24, 27). However, these apparently conflicting findings might reflect heterogeneous hospital-level case-complexity where hospitals that care for increasingly complex patients may be less able to reduce costs and improve quality (24). This potential has given rise to the worry that hospital VBP programs might then exacerbate health disparities by disproportionately penalizing participating hospitals that serve larger proportions of disadvantaged populations (10, 12, 28). Safety-net hospitals, for instance, are more likely to serve patients with higher social risk factors and thus have worse performance measures on average (29). Therefore, hospital-based VBP programs may unintentionally increase financial penalties for social-safety net hospitals (30). Indeed, prior evidence indicates that safe-net hospitals participating in hospital VBP programs perform worse on several quality and cost measures compared with non-safety-net hospitals are thus more likely to be penalized (12, 24, 30–32).

Currently in the U.S., disparities exist in almost all aspects of health and health care, including access to care, quality, and health care utilization and outcomes (6, 33–35). The Institute of Medicine (IOM, now the National Academy of Medicine) in the U.S. defines health care disparities as racial or ethnic differences in the quality of health care that are not due to access-related factors or clinical needs, preferences, and appropriateness of intervention (36). However, health disparities are complex and multidimensional and can occur across various dimensions such as race and ethnicity, biological sex, sexual identity, age, disability, socioeconomic status, and geographic location (8, 37). In the U.S., substantial differences in social determinants of health including low socioeconomic status, poverty, lack of access to care exist along the race and ethnic lines that crucially contribute to disparities and poor health and health outcomes (7). The health and health care differences between groups of people that are closely linked with social, economic, and environmental/geographic disadvantages, which are not well-explained by variations in health care needs, treatment recommendations, or patient preferences, are broadly considered health and health care disparities (6–8). Disparities can be seen across a wide range of health conditions, including heart and cardiovascular diseases, hypertension, diabetes, cancer, asthma, and other acute or chronic health conditions (38).

As the U.S. population becomes more diverse, identifying and addressing health and health care disparities takes the forefront of most critical issues in the country (39). One of the overarching goals of Healthy People 2030 is to achieve health equity, eliminate health disparities, and attain health literacy by individuals to improve the health and wellbeing for the entire population (40). Further, health and health care disparities carry a huge economic burden. Health inequities in general are estimated to account for an approximate value of $320 billion in annual health care spending in the U.S. with a trajectory of reaching $1 trillion or more by year 2040 if left unaddressed (38). If the future projected estimates are reached, the country would face critical challenges related to health care quality, affordability, and access for the entire population that could have major additional consequences for the health and wellbeing of all individuals, especially the historically underserved populations (38). Health care disparities also have critical consequences for quality of life. An estimated $42 billion in lost productivity per year is accrued due to health disparities without even accounting for the economic loss of premature death (38).

Currently, relative to the knowledge of general impacts of hospital based VBP programs on health care quality and cost, comparatively less solid evidence is available on the impact of these programs on health care disparities (24). No study has thus far structurally reviewed and evaluated the collective empirical evidence and implications of the U.S. hospital VBP programs for health care disparities. Theoretically, most value-based programs do not explicitly incentivize equity and may inadvertently increase disparities as a side-effect of the incentive-based payments to providers. It is then imperative to review and analyze the existing evidence, and assess whether the implementation of hospital VBP programs have the potential to worsen disparities in the U.S.

## Methods and results

### Link between VBP and inequality from a conceptual perspective

Researchers and many national health policy advisory organizations such as the National Academies of Sciences, Engineering, and Medicine (NASEM) and Medicare Payment Advisory Commission (MedPAC) have recently analyzed newly available outcomes data and raised serious concerns over the Medicare VBP programs as they are currently structured (11, 41–45). They are particularly concerned about the potential biases of the VBP programs and their implications for health care equity and disparity (46–50). Specifically, these programs are suspected to have penalized hospitals and medical practices that serve patients of predominantly lower socioeconomic status (SES) who tend to live in areas with higher concentrations of poverty and minority population groups (12, 51–55). Some of the biases arose from the budgetary constraints and incentives design inherent in the existing VBP programs, while others were the results of their implementation as providers made self-interested decisions that might not be in the best interest of their patients. Indeed, a misalignment of financial incentives and policy goals (e.g., health equity and quality for all) can result in unintended consequences for socially at-risk population groups (41, 56).

In the post-ACA era of P4P innovation, three distinctive yet related forces of financial self-interest, budgetary constraints, and strategic gaming behaviors created a perfect storm of unintended consequences. First, Congress authorizes an annual budget to fund the operation of the federal government. The various federal agencies can propose reforms and administer them with the consent of the U.S. Congress, but they often proceed under the principle and fiscal constraint of budget neutrality. It is a zero-sum game, in which winners gain at the expense of losers who tend to be providers that treat a disproportionate share of low-income patients with greater social risk factors (41, 55). In this respect, there may be unintended consequences such that providers caring for disadvantaged or low SES patients show worse performance than their counterparts, even after adjusting for between-plan and between-provider disparities (57).

Second, not all the patients under the care of health care providers, particularly in the hospital setting, are equally healthy. Patients of lower SES tend to be sicker and costlier to treat than those who are more educated and affluent (58). However, evidence suggested that the hospital VBP programs implemented do not adequately take patients' clinical and socioeconomic characteristics into consideration when rating quality of care (44, 51). Further, many medical facilities and practices in poor neighborhoods do not have advanced electronic medical record systems and as a result, cannot produce the necessary data to attest to the quality of their services. Others are rated unfavorably simply because they serve predominantly vulnerable patient populations, including minority patients, who are associated with greater social and clinical risk factors that make them more complex and costly to treat on average. Thus, these hospitals are more likely to be financially penalized relative to practices treating patients with lower average social and clinical risks (52, 55).

Third, the CMS's VBP programs, as they are currently structured, encourage medical practices and their providers to avoid poorer and sicker patients and penalize medical providers located in poorer neighborhoods that treat a predominantly minority patient base (12, 45, 59). This behavior of selectively enrolling or accepting patients might create faulty underinvestment in the quality of care for patients with social risk factors (8, 41, 60), and further widen the gap of the already existing disparities between the rich and poor.

Economists have long understood that health care differs from other consumer goods and services because patients lack the necessary information to make rational choices and medical treatments that themselves involve inherent risks and uncertainties (61). Health care consumers are particularly more likely to be disadvantaged both financially and medically because of information asymmetry since providers know far more than their patients who rely heavily on their physicians and other providers to both diagnose and deliver the recommended services (1, 62, 63). These special characteristics of health care and the heavy reliance on health care providers as trusted agents to look after patients' best interests have resulted in a special relationship between them (64, 65). The fiduciary view of this relationship, shared commonly by ethicists and philosophers, sees physicians as having a moral obligation to do the best for their patients. In the opposing view, held mostly by legal experts and economists, undesirable outcomes may occur when physicians and patients disagree on what is best and/or when providers place their own interests ahead of those of the patient.

A new dimension of the mentioned relationship emerges with the additional consideration of patient SES. According to the Fundamental Cause Theory of Link and Phelan (1995), a link exists between SES and health status of poorer and less-educated individuals because SES “embodies an array of resources, such as money, knowledge, prestige, power, and beneficial social connections that protect health no matter what mechanisms are relevant at any given time” (66, 67). In other words, SES functions as a fundamental cause of health inequality because, other things being equal, many lower SES individuals lack resources and knowledge to protect and improve their health. For example, evidence suggests that disparities in cancer survival outcomes between white patients and other racial/ethnic minorities were greater for diseases that are more remediable or amendable to treatment (68). Further, social components such as racism and residential segregation may also serve as a fundamental cause of health inequity (69). Those elements, also called upstream structural factors (e.g., income, social status, etc.) have been increasingly considered to be incorporated in health-related interventions or programs (70). They aim to reform or amend the structural components that could affect health by, for example, redistributing resources, opportunities, etc. (70). Since incentive structures for VBP programs are primarily based on financial penalties and rewards, they tend to potentially worsen disparities because physicians and hospitals serving disadvantaged and minority patients and those with complex health issues are more likely to be penalized due to poorer clinical outcomes that are more associated with the patients' SES than with the quality of treatment.

### Empirical evidence of the link

To examine the empirical effects of the CMS' hospital VBP programs on disparities, we conducted a search by focusing only on peer-reviewed, quantitative, English language, published articles. To select these articles, we first identified relevant ones that were released between 2012, the year HVBP and HRRP programs were both implemented, up to August 1, 2022, by conducting a MEDLINE search using each of the three VBP programs (i.e., HVBP, HRRP, and HACRP) as primary keywords, as well as a combination of secondary terms and phrases. To select secondary keywords in our search, we employed the Equity Framework (71, 72) as a guide. The framework was conceptualized and designed by the Committee on Accounting for Socioeconomic Status in Medicare Payment Programs—NASEM in 2016 to identify social factors that potentially affect performance indicators used in Medicare VBP programs and those influence patients' health outcomes (71, 72). Thus, in conjunction with the primary keywords, the secondary search terms utilized included: “disparities” “equity,” “socioeconomic status,” “dual Medicare/Medicaid eligibility,” “race and ethnicity,” “language barriers,” “gender identity,” “sexual orientation,” “marital status,” “geographic location,” “neighborhood,” “rural/urban location,” and “health literacy.” We also operationalized other constructs related to disparities such as “safety-net status” and “safety-net hospitals” that are widely referenced in health disparity literature.

The first round of search resulted in identifying several key articles evaluating the three VBP programs. We then extended the search to related studies from the key articles' references and their subsequent citations. This was performed by conducting a “backward search” by identifying relevant citations from key papers, and a “forward search” through Google Scholar to pinpoint relevant articles that cited the key articles. Together, the search yielded a combined total of 469 articles as non-unique results. We screened them by their titles and abstracts to determine whether they pertained to the CMS' hospital VBP programs. Finally, 35 articles were chosen for our review after eliminating duplicate studies and those that did not examine the programs' impact on disparities, or were shorter commentaries, or dispatches from the field. We summarized the selected studies and chronologically listed them based on their year of publication (see Table 2).

**Table 2:** Review of studies exploring the effects of medicare hospital VBP programs on health care disparities in the United States.

**Author (year)**	**Hospital VBP program**	**Study design**	**Data sources & settings**	**Study population**	**Primary outcome(s)**	**Main results**	**Does the investigated hospital VBP program(s) potentially exacerbate health care disparities?**
Joynt and Jha (86)	HRRP	Observational	Private + Public	3,282 hospitals	Hospital penalty status under HRRP (high penalties, low penalties, and no penalties)	Major teaching and safety-net hospitals were more likely to be highly penalized compared to nonteaching and non-safety-net hospitals, respectively. The odds of being highly penalized were greatest for safety-net hospitals (adjusted OR = 2.38, 95% CI: 1.91–2.96, ***P*** < 0.001) compared to non-safety-net hospitals.	Yes
Ryan (48)	HVBP	Observational	Public (Medicare)	2,981 hospitals	The HVBP payment adjustment	Hospitals with a greater DSH score were significantly associated with a lower Medicare payment adjustment, affecting expected financial impact more negatively in the first year of the program.	Yes
Dupree et al. (80)	HVBP	Observational	Private + Public	3,030 hospitals	A surgical composite score based on seven surgical performance measures in the HVBP program	Compared to for-profit hospitals, public and nonprofit hospitals had lower composite surgical performance scores by 15.6% and 9.7%, respectively.	Yes
Gilman et al. (83)	HVBP & HRRP	Observational	Public (Medicare & Medicaid)	242 hospitals in California	Financial penalty status under the investigated programs & Total VBP performance scores & The average 30-day risk-adjusted mortality rates for AMI, HF, and pneumonia	Safety-net hospitals were more likely than other hospitals be subject to VBP and HRRP penalties and more likely to experience reductions in payments under the HRRP. Safety-net hospitals were marginally more likely to have a lower process score, and more likely to have a significantly lower patient experience score. Safety-net hospitals were performing slightly better in terms of the average 30-day risk-adjusted mortality rates for AMI, HF, and pneumonia.	Yes
Gu et al. (84)	HRRP	Observational	Public (Medicare & Medicaid)	3,359 short-term acute care hospitals	Excess readmission ratios	Hospitals with a higher proportion of dual-eligible patients, than their counterparts, were more likely to have excessive readmissions and were more likely to have penalties.	Yes
Nagasako et al. (87)	HRRP	Observational	Public (Medicare)	71,793 index admissions involving 59,554 unique patients	30-day all-cause readmission rates for AMI, HF, and pneumonia	Accounting for the census tract-level socioeconomic factors did not significantly influence the average 30-day risk-standardized readmissions rate for each of the target conditions. However, variations in the risk-adjusted performance and readmission rates among hospitals reduced substantially and the overall range of hospital performance in each of the measures was narrower after inclusion of these factors and declined from 6.5 to 1.8 PP for AMI, 14.0 to 7.4 PP for HF, and 7.4 to 3.7 PP for pneumonia.	Yes
Gilman et al. (30)	HVBP & HRRP	Observational	Public (Medicare & Medicaid)	3,022 acute care hospitals participating in VBP and the HRRP	Financial penalty status, and the magnitude of penalties, under both VBP programs & HVBP performance scores & Excess readmission ratios for AMI, HF, and pneumonia under HRRP	Safety-net hospitals were more likely to be penalized compared to non-safety-net hospitals, and they had larger payment penalties under both programs. Safety-net hospitals had worse average process and patient experience scores and they were more likely to receive a reduced payment rate due to HVBP, but less likely to receive VBP bonus payments, when compared to non-safety-net hospitals. Safety-net hospitals had higher readmission ratios for AMI, HF, and pneumonia in comparison to non-safety-net hospitals, and they were at greater risk for receiving a reduced payment rate under the HRRP.	Yes
Gilman et al. (32)	HVBP	Observational	Public (Medicare)	2,695 acute care (673 safety-net and 2,022 non-safety-net) hospitals participating in the VBP program in 2014	Financial penalty status under HVBP & The average VBP process-of-care, patient experience, and mortality (survival) scores & The average 30-day risk-adjusted mortality rates for AMI, HF, and pneumonia	Safety-net hospitals were at greater risk of receiving reduced payments than other hospitals (63% vs. 51%, respectively) and they were less likely to receive bonus payments under the program. Safety-net hospitals were significantly more likely to have a worse process and patient experience scores. Safety-net hospitals' actual overall performance on mortality was slightly better than that of non-safety-net hospitals.	Yes
Herrin et al. (85)	HRRP	Observational	Private + Public (Medicare & Medicaid)	4,073 hospitals	30-day risk-standardized readmission rate	The county in which the hospitals were located accounted for 58% of national variations in hospital readmission rates and county characteristics accounted for almost 48% of the total variation in rates across counties. Specifically, number of Medicare beneficiaries per capita, low education area status, higher numbers of specialists per capita, and hospital beds per capita were all associated with significantly higher readmission rates while higher numbers of nursing homes per capita was associated with lower readmission rates. Safety-net hospitals had higher readmission rates compared to non-safety-net hospitals.	Yes
Rajaram et al. (88)	HACRP	Observational	Private + Public (Medicare)	3,284 hospitals	Hospital penalization status under HACRP	Safety-net hospitals were more likely to be penalized under HACRP (adjusted OR = 1.36, 95% CI: 1.11–1.68, *P* = 0.004) compared to other hospitals. Further, very major teaching hospitals (adjusted OR = 2.61; 95% CI, 1.55–4.39, *P* < 0.001) and major teaching hospitals (adjusted OR, 1.58; 95% CI, 1.09–2.29, *P* = 0.02) were more likely to be penalized under HACRP compared to nonteaching hospitals.	Yes
Shih et al. (90)	HRRP	Observational	Public (Medicare)	255,250 patients from 1,186 hospitals	Excess readmission ratios	Minority serving hospitals, relative to their counterpart non-minority serving hospitals, had a higher likelihood of being penalized (60.8% vs. 32.3%) and having reduced Medicare payments ($112 million vs. $41 million).	Yes
Carey and Lin (76)	HRRP	Observational	Private + Public (Medicare)	All hospitals reimbursed under the inpatient prospective payment system in fiscal years 2013 and 2016	30-day risk-adjusted readmission rates for AMI, HF, and pneumonia	Between fiscal years 2013 and 2016, the readmission rates for all three target conditions were higher in safety-net hospitals compared to other hospitals and they had smaller improvements over time. However, the gap of readmission rates between safety-net and other hospitals narrowed over the first three years of the HRRP.	Yes
Figueroa et al. (82)	HVBP, HRRP, & HACRP	Observational	Private + Public	3,052 hospitals	The size and magnitude of combined penalties (most penalized, moderately penalized, and least penalized) across all three programs in Fiscal year 2015	Large, major teaching, and safety-net hospitals were more likely to be penalized the most by all three programs. Similar trends of penalties were observed for each VBP program independently.	Yes
Sheingold et al. (101)	HRRP	Observational	Private + Public (Medicare & Medicaid)	All hospitals' eligible admissions under HRRP in both 2009 and 2012 fiscal years	30-day readmission rates for AMI, HF, and pneumonia	After accounting for patient-level demographic characteristics (including socioeconomic status, race, and dual eligibility), the odds of risk-adjusted 30-day readmission rates at safety-net hospitals were higher by 8% in 2009 and 9% in 2012. Patients' socioeconomic status accounted for about 25% of the difference in the odds of readmissions. Compared to other categories of hospitals, safety-net hospitals did not experience disproportionately high penalties in the first three years of HRRP.	Yes but modestly
Favini et al. (81)	HRRP & HVBP	Observational	Public (Medicare)	3,016 hospitals	Excess readmission ratio & VBP payment adjustment	Under the HRRP, safety-net hospitals had greater penalties than non-safety-net hospitals (−0.37% vs. 0.28%). Further, safety-net hospitals had average excess readmission ratios above one (1), while non-safety-net hospitals had average excess readmission ratios below one (1).	Yes
Mellor et al. (98)	HRRP	Observational	Private + Public (Medicare)	—	30-day hospital readmission & Readmissions that took place within 31-45 days and 31-60 days of the initial hospital discharge	The HRRP significantly reduced readmission for Medicare patients treated for AMI by 2.5 to 2.8 PP, however, the HRRP did not significantly reduce readmission rates for HF or pneumonia. No evidence that hospitals treat patients with greater intensity, delay readmissions, or alter discharge status in response to the HRRP policy during the studied period. No evidence that hospitals avoided minority patients, patients with lower socioeconomic status, and those with medically complex conditions to lower readmissions for patients with AMI under the HRRP policy.	No
Salerno et al. (100)	HRRP	Quasi-experimental	Public (Medicare & Medicaid)	3,254 hospitals (797 safety-net hospitals, 2,457 non-safety net hospitals)	30-day risk-adjusted readmission	Under the HRRP program, 30-day risk-adjusted readmission rate decreased in both safety-net hospitals (from 17.0% to 13.6%) and non-safety-net hospitals (from 15.4% to 12.7%). The gap in the performance between both hospital groups has reduced over time since the implementation of the program.	No
Thompson et al. (91)	HRRP	Observational	Private + Public (Medicare & Medicaid)	3,229 participating hospitals in the HRRP program during its first five years	Being penalized under the Medicare's HRRP during fiscal years 2013 through 2017	More than half of the hospitals received penalties during all five years. Major teaching hospitals, urban hospitals, and large- or medium-sized hospitals (compared to nonteaching, rural, and small hospitals, respectively) were more likely to receive penalties during all five years of HRRP program. Relative to for-profit hospitals, those publicly-owned and not-for-profit hospitals were less likely to be penalized during the programs all five years. Hospitals caring for a relatively higher proportions of socioeconomically disadvantaged (DSH) or Medicare patients were more likely to receive penalties in all five years. The average HRRP penalties increased modestly to 0.60 percent in fiscal year 2017 from its prior value of 0.29 percent in fiscal year 2013. Medium-sized hospitals and hospitals with the higher Medicare proportions experienced higher increases in penalties over time. Hospitals with higher proportions of socioeconomically disadvantaged patients experienced an increase in penalties, however, this increase was similar across all DSH hospital quartiles.	Yes
Al Mohajer et al. (73)	HACRP	Observational	Public	2,249 hospitals	The mean total HACRP score & Receiving CMS penalties	Teaching hospitals, and hospitals with more staffed beds, longer LOS, or higher CMI were more likely to have higher total HACRP scores and more likely to receive a CMS penalty.	Yes
Bazzoli et al. (75)	HVBP & HRRP	Observational	Private + Public (Medicare)	2,720 hospitals	Total margin for hospital	Public and high-DSH hospitals were found to have greater penalty rates than their counterpart non-high-DSH and not-for-profit and for-profit hospitals (0.33 and 0.43 percent, respectively).	Yes
Chaiyachati et al. (77)	HRRP	Quasi-experimental	Public (Medicare & Medicaid)	58,237,056 patient discharges	30-day readmission rates	Disparities in 30-day readmission rates between Black and white patients within safety-net hospitals were worsened by 0.04 percentage points after implementing the HRRP. However, within non-safety hospitals, disparities between Black and white patients stayed stable.	Yes
Chen et al. (78)	HRRP	Observational	Public (Medicare)	All hospitals qualified for the HRRP program from 2013 to 2016, including hospitals from the Mississippi Delta region (252 counties in 8 states: Alabama, Arkansas, Illinois, Kentucky, Louisiana, Mississippi, Missouri, and Tennessee)	Risk-adjusted 30-day all-cause readmission rates for AMI, HF, and pneumonia	The Mississippi Delta region hospitals performed poorly in terms of readmission rates of all three conditions under HRRP between 2013 and 2016, compared to other Delta state hospitals, and other hospitals participating in HRRP in the U.S. However, much of these variations were attributed to hospital geographic location and the community level factors. Other factors that were independently associated with higher 30-day readmission rates included major teaching hospitals and communities with a higher percentage of Black population.	Yes
Figueroa et al. (93)	HRRP	Observational	Public (Medicare)	6,289,225 patients with the targeted conditions (AMI, HF, pneumonia) in 2,960 hospitals	30-day risk-adjusted readmission rate	Improvement in the 30-day risk-adjusted readmission rate after the HRRP implementation was greater among Black patients than whites (0.45% vs. 0.36%). Also, since the HRRP implementation, trends in readmissions improved, particularly among minority-serving hospitals relative to other hospitals.	No
Gai and Pachamanova (94)	HRRP	Quasi-experimental	Private + Public (Medicare)	34 million hospitalizations in 27 states	30-day readmission rate	Overall, substantial decreases in 30-day readmission were found attributed to the HRRP. Notably, readmissions were reduced more for high-risk patients relative to low-risk patients, particularly those with AMI in hospitals with the greatest share of disadvantaged patients.	No
Gaskin et al. (95)	HRRP	Observational	Private + Public (Medicare)	6,564 zip codes in eight states (New York, New Jersey, California, Florida, Arizona, Colorado, Wisconsin, and North Carolina) with hospital discharges in 2006 and 2013	Number of hospital discharges for targeted conditions (HF, AMI, and pneumonia)	The expected penalty for excess readmissions had a significant, adverse effect on the number of hospital discharges for the three targeted conditions. The negative relationship increased as the proportion of minority patients rose. However, such an effect was not shown for the poverty rate.	Yes, but modestly
Huckfeldt et al. (96)	HRRP	Quasi-experimental	Public (Medicare & Medicaid)	3,263 acute care hospitals	Risk-adjusted 30-day all-cause mortality	AMI mortality trends in Black patients showed an improvement, especially compared to white patients (−1.65% difference-in-differences). HF mortality trends seemed to increase in White patients, but not among Black patients, while for pneumonia, similar mortality trends appeared in both racial groups.	No
Kaplan et al. (97)	HRRP	Observational	Observational	1,745,686 Medicare patients aged ≥65 years, from five states (Arkansas, Florida, Nebraska, New York, and Washington) between 2007-2014	30-day all-cause readmission rates for AMI, HF, and pneumonia	There was a significant decrease in readmission rates, between 2007 and 2014, for all three conditions in both safety-net and non-safety net hospitals. The disparities gap between black and white patients in readmission rates narrowed over time. However, this downward trend of reducing disparities started years before HRRP implementation, in both groups of hospitals.	No
Sankaran et al. (89)	HACRP	Observational	Public (Medicare)	15,470,334 patients from 3,238 acute care hospitals	The number of hospital acquired conditions, 30 day readmissions, and 30 day mortality	Penalized hospitals under the HACRP program were more likely to be big, academic hospitals serving a greater proportion of disadvantaged patients. However, penalization was not related to an overall change in 30-day hospital readmission and certain groups' mortality.	Yes
Wasfy et al. (102)	HRRP	Observational	Public (Medicare)	2,868 acute care hospitals with 12,560,914 discharges	30-day all-cause risk-adjusted readmission rates	For HF, risk-adjusted readmission rates decreased more in high than low dual-eligible patients; however, the opposite result was found for pneumonia, while no significant result was shown for AMI. Compared to low dual-eligible hospitals, high dual-eligible ones had a smaller decrease in the rate change of risk-adjusted readmission rates.	Yes, but modestly
de Lancer Julnes and Choi (79)	HRRP	Observational	Public (Medicare)	124,287 records for Medicare patients who were discharged with HF	Risk-adjusted 30-day and one-year readmissions for patients with HF & Risk-adjusted 30-day and one-year mortality rates for patients with HF	Overall, HRRP resulted in reducing risk-adjusted readmission however, risk-adjusted mortality significantly increased for Medicare HF patients during the penalty phase. Compared to HF patients living in higher income census tracts, those living in lower-income areas had a higher likelihood of risk-adjusted readmission (by 4.8%–5.4%), and mortality (by 5.8%–7.1%). Compared to White patients, non-Whites (Blacks, Hispanics, and other races) had a higher likelihood of risk-adjusted readmission (by 13%), but a lower likelihood of mortality (by 15%)	Yes
Hsu et al. (27)	HVBP & HACRP	Quasi-experimental	Private + Public (Medicare & Medicaid)	618 acute care hospitals (145 safety net hospitals, 473 non-safety hospitals)	Rates of 4 health care-related infections (CLABSI, CAUTI, SSI after colon surgical procedures, and SSI after abdominal hysterectomy)	Overall, the implementations of the HACRP and HVBP programs were not related to improvements in health care-related infections, and disparities in them were not narrowed but stayed overtime.	Yes
Pandey et al. (99)	HRRP	Observational	Public (Medicare)	155,397 patients with acute MI, aged ≥65 years, from 753 hospitals enrolled in the multicenter National Cardiovascular Data Registry Chest Pain-MI Registry	30-day all-cause readmission and mortality rates	Despite a steady decline in 30-day readmission and mortality rates for all patients, this decline for the outcomes did not vary across race (Black vs. non-Black) and hospital performance status (high performing vs. low performing). HRRP was not associated with improvement or worsening of racial disparities, however, racial disparities do still exist in the assessed outcomes.	No
Zogg et al. (92)	HACRP	Observational	Public (Medicare)	695,775 patients from 2,923 hospitals	Risk-adjusted HACRP scores	Mean HACRP scores rose as the proportion of Blacks in a hospital increased, indicating that the HACRP program implementation may negatively affect hospitals serving more racial minorities through penalties.	Yes
Aggarwal et al. (24)	HVBP, HRRP, & HACRP	Observational	Public (Medicare)	3,288 hospitals	Hospitals' penalty-bonus status under each value-based payment program	High-proportion Black hospitals were more likely to be penalized by all three programs.	Yes
Banerjee et al. (74)	HRRP	Quasi-experimental	Public (Medicare)	1,915 hospitals (479 safety-net and 1436 non-safety net hospitals)	Risk-adjusted 30-day all-cause readmission rates for AMI, HF, and pneumonia in safety-net and non-safety-net hospitals & The frequency of readmission penalty among safety-net and non-safety-net hospitals.	After HRRP implementation in 2012, there was a similar trend of decline in readmission rates for the three conditions in safety-net and non-safety net hospitals. HRRP was associated with similar change of the readmission rates of AMI and HF in both hospital groups. However, there was a larger change (decline) in pneumonia readmission rate for safety-net hospitals after HRRP. Safety-net hospitals were more likely to be penalized each year between 2013 and 2016, and they were more likely to be repeatedly penalized and have higher average penalty.	Yes

AMI, Acute Myocardial Infarction; CAUTI, Catheter-Associated Urinary Tract Infection; CLABSI, Central Line-Associated Bloodstream Infection; CMS, Centers for Medicare & Medicaid Services; HF, Heart Failure; LOS, Length of Hospital Stay; SSI, Surgical Site Infection; VBP, Value-Based Purchasing; HVBP, Hospital Value-Based Purchasing Program; HRRP, Hospital Readmission Reduction Program; PP, Percentage Points; TPS, Total Performance Score; DSH, Disproportionate Share Hospital index.

Many of the reviewed studies found that the hospital VBP programs have the potential of further exacerbating health care disparities by penalizing hospitals or facilities that treat a disproportionate share of minority and disadvantaged patients (24, 27, 30, 32, 48, 73–92) (see Table 2). For instance, Hsu et al. explored the impact of the HVBP and HACRP Programs on health care-related infections among 628 acute care hospitals. Their findings revealed that “… given the persistent disparities in health care–associated infection rates, value-based incentive programs currently function as a disproportionate financial penalty system for safety-net hospitals that provide no measurable population-level benefits” (27). Similarly, Chaiyachati et al. examined a cohort of hospitals to investigate whether racial disparities in hospital readmission rates might have worsened between safety-net and non-safety-net hospitals after participating in the Medicare HRRP Program. Their findings revealed that safety-net hospitals that treated predominantly low-income minority patients showed an increase in racial disparities in readmission rates (77).

Furthermore, an observational study of 2,981 hospitals participating in the HVBP Program examined whether hospitals that treated more patients with low SES and had other disadvantages as indicated by the Disproportionate Share Hospital (DSH) index had worse performance outcomes under HVBP (48). Their findings showed that hospitals with a higher DSH index value (signifying a heavier concentration of low-income patients) received lower Medicare payments per admission and more unsatisfactory outcomes under the HVBP than those with a lower DSH index value. Carey and Lin reported that during the HRRP implementation, and between fiscal years (FY) 2013 and 2016, the readmission rates for all three initial target conditions (i.e., AMI, HF, and pneumonia) were higher in safety-net hospitals, at the U.S. national level, compared to other hospitals, and they had smaller improvements over the first 3 years of the HRRP (76). Similarly, Thompson et al. analyzed data for 3,229 participating hospitals in the HRRP program during its first 5 years of implementation (FY 2013 through 2017) (91). They found that major teaching hospitals, urban hospitals, and large- or medium-sized hospitals (compared to non-teaching, rural, and small hospitals, respectively), were more likely to receive penalties during all 5 years of the HRRP program. Further, hospitals caring for a relatively higher proportions of socioeconomically disadvantaged (higher DSH index value), or for Medicare patients, were more likely to receive penalties in all 5 years. Hospitals with higher proportions of socioeconomically disadvantaged patients experienced an increase in penalties, however, this increase was similar across all DSH quartiles (91). During the same period, medium-sized hospitals and hospitals with the higher Medicare population proportions experienced greater increases in penalties over time.

Moreover, in an observational study of 3,284 hospitals, Rajaram et al. evaluated hospital characteristics and hospital penalization status under the HACRP in FY 2015 (88). Their key findings indicate that safety-net hospitals were more likely to be penalized under HACRP compared to other hospitals. Major teaching hospitals were also significantly more likely to be penalized under the program compared to non-teaching hospitals (88). Lastly, Aggarwal et al. evaluated hospitals participating in all three programs of HVBP, HRRP, & HACRP in FY 2019 and identified the hospitals' penalty-bonus status under each Medicare hospital VBP. The primary aim of the study was to compare the high-proportion Black hospitals to other categories of hospitals in terms of received a penalty (or bonus) under each program (24). They report that high-proportion Black hospitals were more likely to be penalized by all three programs compared to other categories of hospitals (24).

Not all the reviewed studies reached the same conclusion and a few of them found little or no worsening impact of hospital VBP programs on health care disparities or reached mixed or paradoxical conclusions (93–102) (See Table 2). For example, Gai and Pachamanova assessed the impact of the HRRP Program on hospital readmission for HF, AMI, and pneumonia as the targeted conditions among disadvantaged and high-risk patients and found a substantial decrease in 30-day readmission overall (94). Notably, hospital readmissions were reduced more for high-risk patients than low-risk patients, especially for those with AMI in hospitals with the greatest share of disadvantaged patients.

Separately, a quasi-experimental study of 797 safety-net hospitals and 2,457 non-safety-net hospitals examined the extent to which HRRP implementation was associated with decreases in 30-day readmission rates among safety-net and non-safety- net hospitals (100). Their findings indicated that 30-day risk-adjusted readmission rates decreased among both safety-net hospitals and non-safety-net-hospitals. They also found that safety-net hospitals experienced more rapid decreases in readmissions which narrowed the gap in performance between the two hospital groups.

Huckfeldt et al. investigated whether 30-day mortality rates rose among older Black and White hospitalized patients after the implementation of HRRP, based on an interrupted time-series analysis of a cohort of 3,263 acute care hospitals (96). They concluded that the VBP policy was not associated with an increase in mortality among Black populations. Specifically, they found that 30-day post-discharge mortality for all conditions did not worsen in older Black patients, relative to their White counterparts, after the initiation of HRRP. They further found that mortality rates for heart conditions improved among Black patients compared to White patients (96). Similarly, Sheingold et al. obtained information on hospital readmission rates from the Medicare Hospital Claims data for FYs 2009 and 2012 and calculated the penalties for FY 2013 of the risk-adjusted readmission rates for the target conditions of AMI, HF, and pneumonia (101). The primary aim of the study was to compare safety-net hospitals (high DSH value hospitals) to other categories of hospitals (lower DSH value hospitals) in terms of the risk-adjusted 30-day readmission rates for the three initial target conditions. The findings portrayed that, at least in the first 3 years of the HRRP, safety-net hospitals did not have disproportionately high penalties, compared to other categories of hospitals.

Together, available evidence suggests varied impacts of CMS's Medicare hospital VBP programs on health care disparities. Many of the reviewed studies found that these VBP programs have a great potential to exacerbate the already existing health care disparities in the U.S. while others found evidence of moderating impacts of hospital VBPs on disparities. Interestingly, albeit inconclusive and inconsistent, we observed that many of the studies that found evidence of worsening disparities were those based on the less stringent, non-experimental observational study designs, while the studies that were based on quasi-experimental designs showed varying results. Further research is needed to produce more conclusive and consistent evidence and to explore the extent to which study design affects the conflicting conclusions from the evaluations of Medicare hospital VBP programs.

## Discussion and future research

Many direct and specific modifications and adjustments to the existing VBP Programs have been suggested to address the observed disparities in access and outcomes associated with this otherwise promising hospital innovation experiment. For example, Damberg and collaborators have proposed a post-adjustment payment approach to decrease the unfair burden of performance-based payment models for physicians and their practices that treat a predominantly poor patient base in California (103). To reduce disparities in the receipt of hospital care, similarly, the remedy of stratifying hospitals into, for example, safety-net and non-safety-net subgroups so as to take into account the measurable differences in the underlying costs of treatment when paying for hospital care has been suggested (100). Further, the peer group-based payment adjustment approach was introduced to the HRRP and implemented beginning in 2019. Early evidence shows that peer grouping resulted in relatively lessening the burden of financial penalties serving more socially disadvantaged patients (104–107). However, the current modification has limitations where it merely reflects on its readmission outcomes and not the social risk factors of its patients, which is the fundamental goal, and it might not be an perfect approach to address health care inequities in the current form (107). In other efforts to address health care disparities in hospital VBP programs, objective and data-driven quality assessment or evaluation can be carried out by adjusting for the respective groups' average quality scores instead of the overall scores for all hospitals. To reduce penalties frequently paid by safety-net hospitals, the stratification of acute care hospitals by the DSH Index (i.e., top 20% vs. the rest) has been suggested as a payment adjustment mechanism for reducing disparities of hospital care reimbursement (100).

Disparities could be potentially reduced through the designs of new incentive payment methods and mechanisms. Originally designed for improving the quality and efficiency of health services delivery, for example, alternative payment models (APMs) are suggested as having the additional effects of alleviating health care disparities attributed to the various hospital VBP contracts (4). Further, there has been an effort to urge alignment in payment models or approaches in the U.S. health care system. In 2015, for instance, the Health Care Payment Learning and Action Network (LAN) was created by the CMS as a collaborative private and public partnership among patients, providers, health plans, government agencies, and other stakeholders to enhance the quality of care and the outcomes, including cost containment. Essentially, by transiting to value-based care away from FFS, it conceives a care delivery system that provides person-centered care, which relies on three pillars: quality, cost-effectiveness, and patient engagement (108). Meanwhile, researchers have recommended long-term interventions that address upstream causes of disparities. For instance, higher reimbursements for primary prevention and disease management services are recommended as a long-run strategy for improving population health and reducing persistent health and health care disparities (109, 110). Further, at the organization and delivery systems level, incentives could be offered to health care institutions and organizations that both improve quality and address racial disparities (111). Equally important, new and innovative payment schemes that can help improve health equity should be studied, tested and promoted by involving clinicians experienced in caring for disadvantaged patient populations and by offering funding opportunities to community health workers, especially those who provide care directly to disadvantaged patients (4, 112).

Another promising research opportunity that has not drawn sufficient attention lies in the development and construction of a more solid conceptual and theoretical foundation for the understanding, and design, of effective VBP programs. Recently, Link, Phelan and Tehranifar have recommended that policy strategies should be based on the insights of, for example, a “theory of fundamental causes,” which postulates that the causes of observed disparities originate from the fundamental differences in population-level access to essential economic and social resources (67). Their insightful observation has led to policy emphasis on programs that support medical progress and new interventions that have the potential of lessening or moderating differences in the allocation of socioeconomic resources such as housing for the poor and homeless, social security and disability benefits, minimum wage laws, and the Head-Start programs for pre-K children.

Recently, the progresses from the field of behavioral economics have led researchers to believe that it is feasible and practical to achieve better health outcomes for socioeconomically disadvantaged by providing them with more and targeted support through creative service contracting with health care providers (113). For example, a reallocation of funds from VBP bonus programs that serve low-risk populations to reward providers serving high-risk groups might be effective in reducing disparities. This resource-allocation approach could help address the issue of disparities attributable to VBP programs without having financial disadvantages. Similarly, the field of health economics, with its emphasis on the unique economic characteristics of health care, suggests that physicians, who play a dual role of diagnosis and treatment, can considerably influence patients' demand for health services (114).

Equally promising, a valuable lesson from the field of information economics, the concept of information asymmetry (or imbalance) between patients and their providers, has been credited for the promotion of health care transparency as a path and mechanism to address disparities. The VBP programs can be modified to provide patients, especially those with higher risks of chronic and life-threatening diseases, with effective and meaningful access to, and utilization of, medical information. This and other information economics inspired innovations may in the near future bring about the addition of patients' electronic access to health records, health education opportunities, and health literacy programs as adjunct interventions alongside the main quality and payment interventions under the overall VBP programs.

## Conclusion

VBP is a complicated program that was initiated for improving health care quality and containing costs. It has the potential to improve equity and reduce disparities in health and health care, but little is presently known about the effects of VBP on access to health care and health disparities. Even less is known about the effects of hospital based VBP programs in general and their impacts on disparities in particular. This review examined relevant literature published between 2012 (the year in which HVBP and HRRP were implemented) and 2022 and found mixed results on the impacts of CMS's Medicare hospital VBP programs on health care disparities. Our results suggest three important implications. First, left to the status quo, CMS's hospital VBP is unlikely to reduce health care disparities and might even exacerbate them. Second, focused evaluation and research are needed to ascertain a congruent and conclusive result on the effects of VBP on health care disparities. Third and most importantly, researchers and policy makers need to work together to explore how VBP can be modified based on the latest evidence to improve equity and reduce disparities while at the same time maintaining the momentum already gained in quality improvement and cost containment.

## Author contributions

HK: writing—original draft. AM, NH, and CC: writing—review & editing. All authors contributed to the article and approved the submitted version.

## Funding

This study was supported by the University of Southern Mississippi for the research project assessing value-based payment programs.

## Conflict of interest

The authors declare that the research was conducted in the absence of any commercial or financial relationships that could be construed as a potential conflict of interest.

## Publisher's note

All claims expressed in this article are solely those of the authors and do not necessarily represent those of their affiliated organizations, or those of the publisher, the editors and the reviewers. Any product that may be evaluated in this article, or claim that may be made by its manufacturer, is not guaranteed or endorsed by the publisher.
